# Correction: Hypersexuality Addiction and Withdrawal: Phenomenology, Neurogenetics and Epigenetics

**DOI:** 10.7759/cureus.c1

**Published:** 2015-09-11

**Authors:** Kenneth Blum, Rajendra D Badgaiyan, Mark S Gold

**Affiliations:** 1 Department of Psychiatry, McKnight Brain Institute , University of Florida,; 2 Department of Psychiatry, and Laboratory of Advanced Radiochemistry, University of Minnesota ,School of Medicine, Minneapolis , MN., USA; 3 Departments of Psychiatry & Behavioral Sciences, Keck School of Medicine of USC, Los Angeles, CA, USA

This article erroneously claims that hypersexuality has been included in the *Diagnostic and Statistical Manual of Mental Disorders, Fifth Edition* (DSM-5). The original, erroneous instances are detailed below, along with the corresponding corrected versions.

The opening sentence of the abstract states, “Hypersexuality is now part of the DSM-V [...].” The corrected sentence reads: “Hypersexuality has been defined as abnormally increased sexual activity.”

The final two sentences of the Introduction & Background section state, “Hypersexuality can now be found in the DSM-V. This major accomplishment required intensive work by Kafka, Reid and associates, Bancroft, and even the World Health Organization, among others [8-11].” The corrected sentences read: “Hypersexuality is not found in the DSM-5. This inclusion, which would be a major accomplishment, continues to require intensive work by Kafka, Reid and associates, Bancroft, and even the World Health Organization, among others [8-11].”

The opening sentence of the Conclusions section states, “While it is important that hypersexuality disorder is now included in the DSM-V [...].” The corrected sentence reads: “While we firmly believe that it is important that hypersexuality disorder is included in the DSM-5 [...].”

